# Idiopathic Multicentric Hyaline Vascular-Type Castleman Disease

**DOI:** 10.1155/2021/6620666

**Published:** 2021-04-15

**Authors:** Adelaide Moutinho, Rita Gamboa Cunha, Sheila Koch Jamal, Marta Meleiro Lisboa, Sandra Tavares

**Affiliations:** Department of Internal Medicine, Hospital de Chaves—Centro Hospitalar de Trás-os-Montes e Alto Douro, Vila Real, Portugal

## Abstract

Castleman disease is a rare lymphoproliferative disorder presenting with localized or disseminated lymphadenopathy and systemic symptoms. It can be categorized clinically as unicentric or multicentric, histopathologically as hyaline vascular, plasma cell, or mixed variant, and etiologically, considering the subtypes based on causative viral agents and associated syndromes. The multicentric type can mimic other haematological malignancies, ranging from asymptomatic to multiple organ involvement. Although its pathophysiology is not well known, the current approved treatments are directed towards interleukin-6, CD-20, and viral agents. The authors present an 82-year-old leucodermic man presented with a 2-week history of constitutional symptoms. Examination revealed pallor, hepatosplenomegaly, and palpable left axillary lymphadenopathy. Investigation showed anaemia, thrombocytopenia, polyclonal hypergammaglobulinemia, hypoalbuminemia, and high acute phase reactants, with image study revealing multiple axillary, mediastinal, inguinal, and pelvic lymphadenopathies. The lymph node biopsy was consistent with hyaline vascular-type Castleman disease without human herpersvirus-8 markers. He started prednisolone with initial improvement evolved poorly on a short term. Castleman disease has a broad spectrum of clinical manifestations, associations, and complications that bring a diagnostic challenge, requiring a multidisciplinary approach. Clinicians should be familiar with its features because proper diagnosis and aggressive targeted treatment are the pillars of proper management of these patients.

## 1. Introduction

Castleman disease (CD) is a heterogeneous group of lymphoproliferative diseases affecting single or generalized lymph nodes which can be classified clinically as unicentric or multicentric and histologically as hyaline vascular, plasma cell variant, or mixed type [1–4]. The hyaline variant, the most frequent one, is characterized by capillary proliferation with small hyaline vascular follicles and usually has a benign clinical course. The multicentric presentation can be linked to human herpesvirus-8 (HHV-8) infection, associated with POEMS, or be idiopathic, the latter recently subclassified in TAFRO-associated idiopathic multicentric Castleman Disease (iMCD) [1, 3, 5–7]. The authors report a case of a Portuguese patient with iMCD presenting with a two-week history of constitutional symptoms, hepatosplenomegaly, and a single palpable left axillary lymphadenopathy, with histology confirming hyaline vascular variant.

## 2. Case Presentation

**Figure 1:**
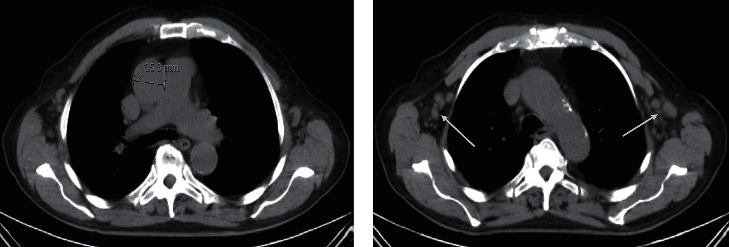
(a) CT scan showing mediastinal lymphadenopathy measuring 35.3 mm (marker); (b) CT scan showing bilateral axillary lymphadenopathies (arrows).

An 82-year-old leucodermic man, with arterial hypertension with hypertensive cardiopathy and no other known end-organ damage, controlled with olmesartan 20 mg id, heart failure with preserved ejection fraction, class II New York Heart Association (NYHA), benign prostate hyperplasia medicated with *Serenoa repens* 160 mg id, and hyperuricemia controlled with allopurinol 300 mg id, presented to our emergency care unit. He had a 2-week history of low fever (axillary temperature 38°C), shivers, night sweats, fatigue, dry cough, and weight loss of 4 kg, with no response to paracetamol 1000 mg that he took three times a day for 3 days. His physical examination revealed a pale skin, a nontender, dull, 4 cm-diameter left axillary lymphadenopathy, nontender hepatomegaly palpable 8 cm below the right costal margin, and nontender palpable splenomegaly. He had stable vitals and normal pulmonary and cardiac examination, with no signs or symptoms of peripheral neuropathy. Laboratory findings revealed anaemia with haemoglobin of 9 g/dL, normocytic (mean corpuscular volume 87,0 fL; normal range (87–103)), normochromic (mean corpuscular haemoglobin 29,8 pg; normal range (27–33)), thrombocytopenia 129.000/*μ*L, and a normal white cell count of 5.000/*μ*L. His reticulocyte count was 1.21% with a reticulocyte index of 0.49 indicating medullary hypoproliferation, and his blood smear confirmed the normochromic normocytic red cells and thrombocytopenia with no further morphological changes. Regarding the coagulation study, he had a prothrombin time of 16.9 seconds, with an international normalized ration of 1.21, a partial thromboplastin time of 31.1 seconds, and normal-range fibrinogen (205 mg/dL) and d-dimer (0,48 *μ*g/mL). There was no deficit of B12 vitamin, folic acid, or iron, with high acute phase reactants: ferritin 762 ng/mL, sedimentation rate 132 mm/1^st^ hour, and C-reactive protein 6.87 mg/dL. His serum creatinine level was 1.3 mg/dL with a creatinine clearance of 63 mL/min, with 399 mg of protein in the 24-hour urine and normal urine sediment. The protein electrophoresis showed a polyclonal hypergammaglobulinemia with an albumin of 2.3 g/dL and total proteins of 4.3 g/dL. The electrolytes, hepatic enzymes, alkaline phosphatase, lactic dehydrogenase, and bilirubin were within the normal range. He had negative human immunodeficiency virus (HIV) 1 and 2 antibodies, negative anti-hepatitis C virus, and was not immune to hepatitis B virus. The immunologic study with antinuclear antibodies (ANAs) and anticytoplasm antibodies and angiotensin-converting enzyme was negative. The image findings obtained by the computerized tomography (CT) scan revealed homogeneous hepatomegaly with a bipolar diameter of 180 mm and splenomegaly with 146 mm of diameter without focal lesions and numerous lymphadenopathies located in multiple lymph node groups: left axillary, with the largest measuring 21.5 mm in diameter, mediastinum, supraclavicular, pelvic, and inguinal (Figure 1). There were no pleural or peritoneal effusions.

**Figure 2:**
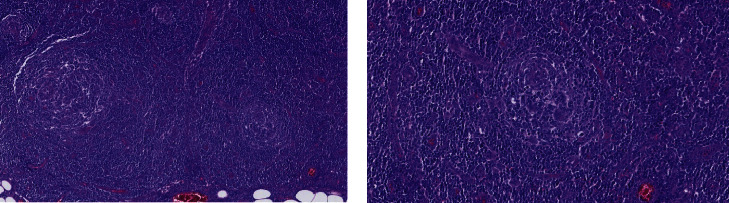
Lymph node biopsy showing features of Castleman disease, hyaline vascular type. (a) HE coloration, 10x amplification. Numerous small follicular structures, follicular dendritic type cells, and blood vessels, some with hyaline walls. (b) HE coloration, 20x amplification. Concentric layers of lymphocytes around the follicles, the mantle zone.

The bone marrow biopsy and myelogram revealed normocellularity, with hyperplasia of all series without lymphoproliferative involvement, with increased reticulin staining. The axillary lymph node biopsy (Figure 2) revealed numerous small follicular structures, with small centres containing CD21-positive follicular dendritic-type cells, endothelial cells, and CD20-positive small mantle lymphocytes arranged in concentric rings. The interfollicular areas contained rare well-differentiated CD138-positive plasmocytes. There was not any positivity for HHV-8 in any of the identified cells. Blood vessels with some hyaline walls were seen in the germinative centres. This appearance is highly suggestive of Castleman disease hyaline vascular type, and a diagnosis of iMCD hyaline vascular variant was made. Regarding the recent classification accepted by the Castleman Disease Collaborative Network, we can retrospectively assume the diagnosis of iMCD-TAFRO type, by compiling all histopathological criteria (typical lymph node pathology and negativity for HHV-8), three major criteria (fever >38°C, reticulin fibrosis, and organomegaly), and one minor criteria (hyperplasia of megakaryocytes within bone marrow biopsy) [1].

The patient started treatment with prednisolone 1 mg/kg/day with clinical, biochemical, and haematological improvement, as a bridge to improve clinical and hematologic status, waiting to start immunotherapy which was planned with anti-interleukin-6 (IL-6) therapy, namely, tocilizumab. However, he evolved with pulmonary oedema secondary to acute heart failure, pancytopenia, and end-organ damage without haemorrhage, coagulopathy, or intravascular disseminated coagulation criteria, without any response to the therapy imposed, deceasing 20 days after the final diagnosis.

## 3. Discussion

Since the first case report published by Dr. Benjamin Castleman in 1954, the term Castleman disease has been applied to several different lymphoproliferative disorders. This case report presents an elder man with CD classified clinically as multicentric, etiologically as idiopathic, and histologically as hyaline vascular variant.

The multicentric CD occurs normally in patients in the sixth decade of life, and it can occur in association with HHV-8 in HIV-positive and -negative patients and POEMS syndrome, or it can be idiopathic, subclassified in TAFRO associated or NOS, the latter when the criteria for TAFRO are not met [1–3, 6, 7]. Like the HHV-8-associated multicentric CD, the idiopathic variant shows histological features of both hyaline vascular and plasma cell variant CD. However, contrary to the case presented where the hyaline vascular type stands out, the idiopathic multicentric CD generally presents with marked plasmacytosis and a lesser degree of vascular proliferation and hyalination [5, 8], which are the typical histological patterns of plasma cell variant CD.

The similarity of the presentation of idiopathic multicentric CD with other lymphoproliferative disorders allied to the rarity of CD, the age of the patient, the clinical symptoms and signs, and laboratory findings induced the initial investigation of haematological malignancies with a main focus on lymphomas. On the other hand, the analysis of the lymph node biopsy had to be reviewed after the consideration of CD as a possible diagnosis, reminding us that the pathological findings with hematoxylin and eosin (HE) coloration are not entirely specific, since they can be seen in other reactive and neoplastic conditions [2], making the diagnosis even more challenging and, in this particular case, delaying it.

On the contrary that unicentric CD in which surgical treatment with or without radiotherapy is usually curative with a good prognosis, the optimal management of multicentric CD has not been well established and the outcome is clearly less favourable [1, 3, 7]. The latter guidelines for idiopathic Castleman disease presume the evaluation of the severity of the disease to decide the best treatment approach, being anti-IL6 monoclonal antibody the first line of treatment, with siltuximab or, as an alternative, tocilizumab [1, 3, 6, 9]. Rituximab, a monoclonal anti-CD20 antibody, is proven to be highly effective in HIV-positive multicentric CD, but it can be used as well in HIV negative patients [3], as an alternative to anti-IL6 monoclonal antibodies. Corticosteroids may be used as monotherapy in patients with more indolent diseases or as a symptom controller, but as well combined with immunotherapy with anti-IL6 or anti-CD20 monoclonal antibodies [1, 3, 9], this latter option being the one chosen to treat the patient presented. However, the immediate unavailability of the immunotherapy and the poor evolution of our patient made it impossible to perform.

The prognostic factors regarding CD are not well known. However, as it happens with other lymphoproliferative disorders, older age is associated with poorer prognosis [3]. Likewise, some studies report splenomegaly and low albumin levels as factors with worse prognosis [3]. POEMS syndrome and renal involvement with reduced creatinine clearance are independent prognostic factors [10]. The TAFRO subtype of iMCD is an aggressive clinical entity associated with multiorgan involvement, with quick and unfavourable evolution without proper treatment [1]. Considering this, and reviewing our case, there were some obvious indicators of a bad outcome.

In conclusion, although idiopathic multifocal CD currently has evidence-based consensus diagnostic and treatment guidelines, it should be considered as a differential diagnosis more often when patients who present with generalized lymphadenopathy and systemic symptoms, and the diagnosis must be properly made. Initiatives such as the unique Castleman Disease Collaborative Network are crucial to maximize the endeavours of research and data sharing of this rare disease, promoting its understanding and proper management of similar cases, and the expansion of knowledge of this entity.

## Data Availability

The clinical data used to support the findings of this study are included within the article.
